# The Relevant Hormonal Levels and Diagnostic Features of Polycystic Ovary Syndrome in Adolescents

**DOI:** 10.3390/jcm9061831

**Published:** 2020-06-11

**Authors:** Elena Khashchenko, Elena Uvarova, Mikhail Vysokikh, Tatyana Ivanets, Lyubov Krechetova, Nadezhda Tarasova, Iuliia Sukhanova, Fatima Mamedova, Pavel Borovikov, Ivan Balashov, Gennady Sukhikh

**Affiliations:** 1 FSBI “National Medical Research Center for Obstetrics, Gynecology and Perinatology Named After Academician V.I. Kulakov” Ministry of Healthcare of the Russian Federation, 4, Oparina Street, 117997 Moscow, Russia; e_uvarova@oparina4.ru (E.U.); mikhail.vyssokikh@gmail.com (M.V.); t_ivanets@oparina4.ru (T.I.); l_krechetova@oparina4.ru (L.K.); compasstar@gmail.com (N.T.); iu.sukhanova@outlook.com (I.S.); f_mamedova@oparina4.ru (F.M.); p_borovikov@oparina4.ru (P.B.); i_balashov@oparina4.ru (I.B.); g_sukhikh@oparina4.ru (G.S.); 2 Department for Obstetrics, Gynecology, Perinatology and Reproduction, Sechenov First Moscow State Medical University, Trubetskaya str. 8, bld. 2, 119991 Moscow, Russia; 3 A.N. Belozersky Research Institute of Physico-Chemical Biology MSU, Leninskye Gory, House 1, Building 40, 119992 Moscow, Russia; 4 Institute of Molecular Medicine, Sechenov First Moscow State Medical University, Trubetskaya str. 8, bld. 2, 119991 Moscow, Russia

**Keywords:** polycystic ovary syndrome, adolescents, hyperandrogenism, free androgen index, anti-Müllerian hormone, leptin, androstenedione, testosterone, ovarian volume, threshold

## Abstract

Relevance: The clinical picture of polycystic ovary syndrome (PCOS) is extremely polymorphic, especially in adolescence. At the same time, the diagnostic criteria of PCOS in adolescence are still under discussion, and the hormonal parameters, including anti-Mullerian hormone range and hyperandrogenism, are not determined. The aim of the present study was to characterize the pivotal clinical and hormonal features of PCOS in adolescents and to establish the age-specific thresholds of the most essential hormonal parameters. Design: A case-control study. Methods: The study included 130 girls with PCOS according to the complete Rotterdam criteria, aged 15 to 17 years. The control group consisted of 30 healthy girls with a regular menstrual cycle of the same age. A complete clinical and laboratory examination, hormonal assays, and ultrasound of the pelvic organs were performed. The serums anti-Mullerian hormone (AMH), follicle-stimulating hormone (FSH), luteinizing hormone (LH), LH/FSH, prolactin, estradiol, 17α-OH progesterone (17α-OHP), androstenedione, testosterone (T), dehydroepiandrosterone sulfate (DHEAS), sex hormone-binding globulin (SHBG), leptin, and free androgen index (FAI) were analyzed. The diagnostic accuracy of AMH, FAI, LH/FSH, T, and androstenedione levels in predicting PCOS in adolescents was established using a logistic regression model and calculating area under the receiver operator characteristic (ROC) curve (AUC). Results: The serum levels of LH (9.0 (5.4–13.8) vs. 3.7 (2.5–4.7) IU/L; *p* < 0.0001), LH/FSH (1.6 (1.0–2.3) vs. 0.7 (0.5–1.1); *p* < 0.0001), 17α–OHP (4.1 (3.2–5.1) vs. 3.4 (2.7–3.8) nmol/L; *p* = 0.0071), cortisol (464.0 ± 147.6 vs. 284.0 ± 129.7 nmol/L; *p* < 0.0001), prolactin (266.0 (175.0–405.0) vs. 189.0 (142.0–269.0) mIU/L; *p* = 0.0141), T (1.9 (1.2–2.5) vs. 0.8 (0.7–1.1) nmol/L; *p* < 0.0001), androstenedione (15.8 (11.6–23.2) vs. 8.3 (6.5–10.8) ng/mL; *p* < 0.0001), AMH (9.5 (7.5–14.9) vs. 5.8 (3.8–6.9) ng/mL; *p* < 0.0001), FAI (5.5 (2.8–7.0) vs. 1.6 (1.1–2.3); *p* < 0.0001), SHBG (37.0 (24.7–55.5) vs. 52.9 (39.0–67.6) nmol/L; *p* = 0.0136), DHEAS (6.8 ± 3.2 vs. 5.1 ± 1.5 μmol/L; *p* = 0.0039), and leptin (38.7 ± 27.1 vs. 23.7 ± 14.0 ng/mL; *p* = 0.0178) were significantly altered in the PCOS patients compared to the controls. Multivariate analysis of all studied hormonal and instrumental parameters of PCOS in adolescents revealed as the most essential: AMH level > 7.20 ng/mL, FAI > 2.75, androstenedione > 11.45 ng/mL, total T > 1.15 nmol/L, LH/FSH ratio > 1.23, and the volume of each ovary > 10.70 cm^3^ (for each criterion sensitivity ≥ 75.0–93.0%, specificity ≥ 83.0–93.0%). The diagnostic accuracy of PCOS determination was 90.2–91.6% with the combined use of either four detected indexes, which was significantly higher than the use of each index separately. The accuracy of PCOS diagnostics reached 92% using AMH and leptin concentrations when the value of the logistic regression function [85.73 − (1.73 × AMH) − (0.12 × Leptin)] was less than 70.72. Conclusions: The results of the study estimate the threshold for AMH, FAI, androstenedione, testosterone, LH/FSH, and ovarian volume, which could be suggested for use in the PCOS diagnostics in adolescents with a high sensitivity and specificity. Moreover, the combination of either four determined indexes improved the diagnostic accuracy for the PCOS detection in adolescents.

## 1. Introduction

Polycystic ovary syndrome (PCOS) among adolescents occurs in 2.2–7.5% of the population and up to 68% in patients with menstrual irregularities and hirsutism [1,2]. Early recognition and management of the disease at the time of its manifestation and before it starts the stage of long-term reproductive and cardiovascular complications is of great importance.

Currently, the diagnostic criteria for PCOS in adolescents are the subject of discussion [3]. The 2018 guideline of Australian National Health and Medical Research Council of Australia (NHMRC) supported by a partnership with ESHRE and the American Society for Reproductive Medicine, 2015 recommendations of the American Association of Clinical Endocrinologists (AACE), the American College of Endocrinology, the Androgen Excess and PCOS Society (AES), as well as 2014 recommendations of the European Society of Endocrinology are designed primarily for the management of adult patients. It is generally accepted that the guidelines require reconsideration for patients in adolescence [1,3,4].

The clinical picture of PCOS is extremely polymorphic. Symptoms of PCOS in adolescence may be similar to those accompanying normal puberty [1,2]. Irregular anovulatory cycles are physiological for adolescents during the several first years after menarche. Indeed, 85% of the cycles are anovulatory in the first year after menarche, and this tendency continues after three years for 59% of the cycles [5,6]. Moreover, aberrant rhythms of gonadotropin releasing hormone pulses, physiological hyperinsulinemia, and functional hyperandrogenism, including mild hirsutism and acne, are also signs of normal puberty [4,7]. Therefore, mild hirsutism alone and isolated acne and/or alopecia in adolescence cannot be considered as clinical evidence of hyperandrogenism (HA) [7].

Hormonal profile and biochemical HA measurements in adolescents have their own gaps, since there is lack of data on reference hormonal intervals for girls with normal puberty, and the results in different laboratories vary widely [1,2,3]. Indeed, no clear cutoff values for total and free testosterone levels, same as for AMH concentrations, exist in adolescents.

One of the most preferred methods of HA evaluation is calculation of free androgen index (FAI) by the ratio of total testosterone (T) and sex hormone-binding globulin (SHBG); however, there is no threshold established in adolescents [3,8] Although it is ideal to use high quality assays such as liquid chromatography–mass spectrometry and extraction/chromatography immunoassays for accurate T assessment, these are not everywhere available and are technically complex. At the same time, rather widespread and still currently used by most practitioners, immunochemiluminescence assays are not recommended because of poor sensitivity and accuracy [1]. A decrease of the SHBG level itself is one of the markers of PCOS, interconnected with insulin resistance and HA [8,9]. The AES work group substantiated the determination of androstenedione level for the HA diagnosis, which is a testosterone’s predecessor and is not bound to SHBG. Its direct radiometric or enzyme-linked assays are more specific and sensitive compared with those for testosterone [9,10]. However, HA determination in adolescents is often difficult, since hormonal levels in girls with PCOS are frequently within the standard values for adults [4,11,12].

One of the common diagnostic criteria for PCOS is polycystic ovarian morphology according to ultrasound data [5,13]. It is well known that the definition of polycystic ovarian morphology in adolescence with transabdominal approach is complicated, particularly in obese patients [10,11]. It was proposed to use the anti-Mullerian hormone (AMH) level as a non-invasive screening test for the diagnosis of PCOS and in addition to the ultrasound parameter, though it has not been validated for girls [4,10]. However, according to the 2016 analysis, the use of AMH level ≥ 7.03 ng/mL gives only 50.0% and 70.8% specificity and sensitivity, respectively, in the diagnosis of PCOS in young patients [11].

Thus, despite numerous studies, there are no approved diagnostic criteria for the disease in adolescence and threshold values for hormonal parameters.

Aim of the current study: to characterize the pivotal clinical and hormonal features of PCOS in adolescents and to establish the age-specific thresholds of the most essential hormonal parameters.

## 2. Materials and Methods

The study included 130 girls aged 15 to 17 years (16.0 (15.0–17.0)), presenting complete Rotterdam PCOS diagnostic criteria (oligo-/amenorrhea; clinical and/or biochemical signs of HA; polycystic ovaries detected by ultrasound). The additional inclusion criteria were: the onset of menarche at least 2 years prior; the absence of other endocrine diseases; absence of drug administration over 3 months preceding the study, including oral combined contraceptives; informed consent of the patient and her legal representative for participation in the research study.

The exclusion criteria were: an aggravation of chronic or acute somatic and/or infectious disease; mental illnesses; inherited syndromes and congenital malformations; hyperprolactinemia; congenital dysfunction of the adrenal cortex; thyroid disorders; Cushing syndrome and disease; tumors of the pelvic organs.

The control group consisted of 30 healthy adolescent girls of the same age (16.0 (15.0–17.0)) with regular menstruations and without gynecological and endocrine pathology. The control group consisted of 30 healthy adolescent girls of the same age (16.0 (15.0–17.0)) without gynecological and endocrine pathology. The girls in the control group had normative weight (55.5 (50.0–62.0) kg) and BMI values (20.2 (18.4–21.8) kg/m^2^). All girls had regular menstrual cycles. The duration of menstrual bleeding ranged from 4 to 8 days. The majority of girls (31, 96.9%) had moderate menstrual bleeding. In 12 (37.5%) girls, menstruations were slightly painful in the first one/two days, which did not require analgesics and did not decrease the girls’ quality of life.

The study was conducted in accordance with the Declaration of Helsinki, and the protocol was approved by the Ethics Committee for Biomedical Research at the National Medical Research Center for Obstetrics, Gynecology and Perinatology named after Academician V.I. Kulakov” Ministry of Healthcare of the Russian Federation, Moscow, Russia (Project identification code № 13, 6 December 2013).

All participants underwent general clinical examination, detailed medical history, complaints, anthropometric indicators (height, body mass index (BMI), waist to hip circumference ratio (WC/HC)), assessment of hirsutism, and the degree of puberty.

The biochemical and lipid profiles of blood were determined for all participants according to the main indicators: total cholesterol, triglycerides (TG), low-density lipoproteins (LDL), high-density lipoproteins (HDL), atherogenic index, and highly sensitive C-reactive protein (CRP). Analyses were performed on automated biochemical analyzers BA-400 and A-25 using photometric and turbidimetric methods and reagents from Biosystems (Spain, Barcelona). An oral glucose tolerance test (OGTT) was performed 12–16 h after the last meal. Glucose and immunoreactive insulin (IRI) levels were measured in venous blood on an empty stomach and the second evaluation 120 minutes after taking 75 g of glucose. The Homeostasis Model Assessment of Insulin Resistance (HOMA-IR) was calculated. The Visceral Adiposity Index (VAI) was used to indirectly estimate the volume of abdominal adipose tissue:

VAI = (WC ÷ (36.58 + (1.89 × BMI)) × (TG ÷ 0.81) × (1.52 ÷ HDL),(1)

where WC (waist circumference) is measured in cm, BMI in kg/m^2^, TG and HDL in mmol/L.

Levels of luteinizing hormone (LH), thyroid-stimulating hormone (TSH), follicle-stimulating hormone (FSH), dehydroepiandrosterone sulfate (DHEAS), androstenedione, prolactin (Prl), estradiol (E2), cortisol, testosterone (T), and sex hormone binding globulin (SHBG) were measured on days 2–4 of spontaneous menstrual cycle or gestagen-induced menstruation-like reaction. Hormonal assays were carried out by electro- and immunochemiluminiscent methods on the automatic analyzers Cobas e 411 (F. Hoffmann-La Roche, Basel, Switzerland), Immulite 2000, and Immulite 1000 (Siemens, Los Angeles, USA) using reagents of the same companies. The immunoassays were standardized via mass spectrometry assays (isotope dilution-gas chromatography/mass spectrometry (ID-GC/MS)) according to the instructions by Roche Diagnostics. Concentrations of AMH and 17-OH progesterone were measured by the enzyme-linked immunoassay on the DYNEX DSX System analyzers and using the Diagnostic Products Corporation (DPC) system on the Immulite device (DYNEX Technologies, VA, USA).

Concentration of leptin was measured using the multiplex assay kit (Milliplex MAP Magnetic Bead Panel 1, Merck KGaA, Darmstadt, Germany) in blood plasma samples using a Luminex 200 flow analyzer (Luminex, Austin, TX, USA) according to the manufacturer’s protocol.

All girls underwent ultrasound examination of pelvic organs as well as mammary and thyroid glands on days 3–5 of a spontaneous or gestagen-induced menstrual cycle. The study was performed on a Vivid-q ultrasonic device of GE HEALTHCARE company (GE Healthcare, Chicago, IL, USA) using a linear and convex probe with a frequency of 1.8–6.0 MHz. The study was conducted with a full filled bladder by the transabdominal approach. The sonographic features including length, width, and thickness (L, W, T, consequently) of left and right ovaries and uterine (U) measurements (L, W, T) were described, and the ovarian volume (OV) was calculated. The ovarian to uterine index (OUI) was established according to the formula:

OUI = (OV_left_ + OV_right_) ÷ (2 × UT), (2)

where OV is measured in cm^3^, and UT (thickness of the uterus) is in cm.

Statistical data analysis was performed using the Statistica 8 software from StatSoft Inc. The categorical variables were evaluated with the χ² test. Comparison of variables with a normal distribution was performed by parametric Student’s t-test, and a mean value (M) and a standard deviation (SD) were calculated. Parameters that did not fit normal distribution were analyzed using a median (Me) and an interquartile range and the Mann–Whitney U-test. The adjusted odds ratio (OR) with a 95% confidence interval (CI) were evaluated using logistic regression methods to analyze the influence of various risk factors. The diagnostic accuracy and cutoff values were assessed by multivariate analysis with the logistic regression models using receiver operator characteristic (ROC) curves and calculating the area under the curve (AUC).

## 3. Results

Among analyzed risk factors of PCOS development, the most significant was the mention of oligomenorrhea and/or PCOS and/or endocrine infertility before pregnancy in a girl’s mother, which increases the risk of the disease in a girl up to five times (*p* = 0.0157, odds ratio (OR) = 4.97; 95% confidence interval (CI) = 1.33; 18.53). Besides, repeated irregular menstrual cycles with delays for more than 90 days after menarche results in three times increased risk of PCOS development in a girl (*p* = 0.0092, OR = 2.98; 95% CI = 1.28; 6.91). Other risk factors such as somatic, infectious, and allergic diseases in childhood appeared to be not significant.

According to a physical examination, patients with PCOS compared with the control group had higher BMI (22.4 (19.9–27.2) vs. 20.2 (18.4–21.8) kg/m^2^, *p* = 0.0002), WC (75.0 (69.0–85.0) vs. 66.0 (62.0–70.0) cm, *p* = 0.0003), and hips circumference (HC) (98.0 (92.0–103.0) vs. 92.0 (89.0–95.0) cm, *p* = 0.0019). Multivariate analysis confirmed that BMI (*p* = 0.0156, OR = 1.08; 95% CI = 1.01; 1.56) and WC (*p* = 0.0237, OR = 1.11; 95% CI = 1.01; 1.19) are significant risk factors for developing PCOS.

Hirsutism was detected in the majority of girls with PCOS (92; 70.8%) (on the upper edge of the lip, the chin, around the nipples, along the midline of the abdomen, on the inner thighs), while this sign was not marked in the controls. The majority of patients with PCOS (77; 59.2%) complained about acne and greasy skin, while there were very few girls with such complaints in the control group (4; 13.3%) (*p* < 0.0001, χ^2^ test).

The patients with PCOS did not differ from the controls by the age of menarche. The comparative analysis revealed that the majority of patients with PCOS (96; 73.8%) had the irregular menstruations from the menarche. In 24 (18.4%) patients with PCOS, menstruations were regular at once, and in 10 (7.7%) patients, the menstrual cycle established within the first year after the menarche. On the contrary, in the control group, the menstruation cycle was established from the first one in the majority of girls (21, 70.0%; *p* < 0.0001, χ2 test), and in the third part, the regular menstruations were established during the first year after the menarche (9, 30.0%; *p* = 0.0007, χ2 test).

At the time of the examination, oligomenorrhea was noted in 67 (51.5%) patients with PCOS, including menstruation delays followed by abnormal uterine bleeding in 17 (13.1%) patients. Amenorrhea was recorded in 43 (33.8%) girls, and primary amenorrhea was registered in 5 (3.8%) patients.

Blood lipid profile and biochemical analysis did not reveal significant differences in the studied parameters between the groups, except the higher total protein, direct bilirubin, and magnesium levels in the PCOS group. Despite the absence of relevant differences in lipids and insulin levels, PCOS patients compared to the control group were characterized with a higher visceral adiposity index (VAI) (1.0 (0.6–1.6) vs. 0.7 (0.5–1.0), *p* = 0.0461), which indicates an increased risk of cardio-metabolic disorders in patients with PCOS already in adolescence (Table 1).

Hormonal profile analysis showed that parameters listed below significantly differed between the PCOS and the control groups, though half of them were within the reference laboratory values (see Table 2).

The study allowed us to identify positive correlations between FAI and BMI (r = 0.583; *p* = 0.013), WC (r = 0.517; *p* = 0.027), TG (r = 0.425; *p* = 0.031), and VAI (r = 0.505; *p* = 0.029) in the PCOS patients. None of the revealed correlations were significant in the control group (*p* > 0.05). Thus, a positive correlation between HA and visceral adiposity hallmarks is typical for adolescent girls with PCOS but not for the healthy girls. Considering association of FAI and BMI in PCOS patients, we conducted analysis of HA status in the PCOS group stratified in two subgroups depending on the presence of excessive weight. We revealed that the subgroup of overweight PCOS girls (BMI>25 kg/m^2^, *n* = 30) differed from normal-weight PCOS girls (BMI < 25 kg/m^2^, *n* = 100) not only by dyslipidemia and atherogenic lipid profile (TG 1.11 ± 0.56 vs. 0.78 ± 0.34, *p* = 0.002; VAI 1.51 ± 0.76 vs. 0.86 ± 0.43, *p* = 0.001; HOMA-IR 5.97 ± 5.41 vs. 2.56 ± 1.48, *p* = 0.001; Leptin 52.22 ± 26.55 vs. 29.19 ± 23.42, *p* = 0.001, hereinafter ANOVA test) but also in more pronounced HA according to the increased level of FAI (7.02 ± 4.33 vs. 4.37 ± 2.70, *p* = 0.002) and decreased SHBG (34.45 ± 25.47 vs. 47.29 ± 20.67, *p* = 0.021) in the overweight group.

The results of the study in the PCOS group show that the leptin level in adolescent patients was highly correlated with body weight (r = 0.637; *p* = 0.003), BMI (r = 0.705; *p* = 0.001), WC (r = 0.679; *p* = 0.002), HC (r = 0.667; *p* = 0.002), and insulin level (r = 0.529; *p* = 0.046), but this was not the case in the control group (*p* > 0.05 for all parameters). The only significant correlations in the control group were for leptin and cholesterol (r = 0.961; *p* = 0.039). 

**Figure 1:**
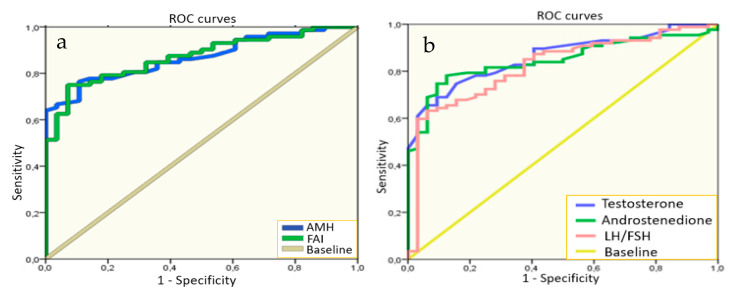
Receiver operator characteristics (ROC) curves for the hormonal levels in predicting PCOS: (**a**) anti-Mullerian hormone (AMH) area under the curve (AUC) = 86.9%, free androgen index (FAI) AUC = 87.1%; (**b**) TestosteroneAUC = 86.1%, androstenedione AUC = 84.7%, luteinizing hormone (LH)/ follicle-stimulating hormone (FSH) AUC = 82.1%; *p* < 0.05 for all indicators.

We performed the multivariate analysis for the search of the hormonal level cutoffs for the most significant indicators in PCOS diagnosis in adolescents to use in practice. The results of these tests revealed that the levels of AMH > 7.20 ng/mL and FAI > 2.75 (Figure 1) showed the highest sensitivity (76.0% and 75.0%) and specificity (89.0 and 93.0%, respectively) for PCOS diagnostics in adolescence. Moreover, we determined that levels of testosterone > 1.15 nmol/L, androstenedione > 11.45 ng/mL, and LH/FSH ratio > 1.23 also showed high sensitivity of 63.2–78.2% and specificity of 84.4–93.7% in PCOS diagnosis in the studied sample of girls.

Comparative analysis of the ovarian structure using 2D ultrasound supported that the PCOS group compared to controls was characterized with higher OV and ovarian to uterine index (OUI), higher number of antral follicles in the slice, and smaller size of minimum and maximum antral follicles (see Table 3).

Multivariate analysis confirmed that, among sonographic parameters, the most significant in the PCOS diagnosis in adolescents were an indicator of the average OV (with the value > 10.70 cm^3^, sensitivity 83.0%, specificity 83.0%, AUC = 84.8%, *p* < 0.05) and OUI (with OUI >3.95, sensitivity 81.0%, specificity 83.0%, AUC = 83.7%, *p* < 0.05).

**Figure 2:**
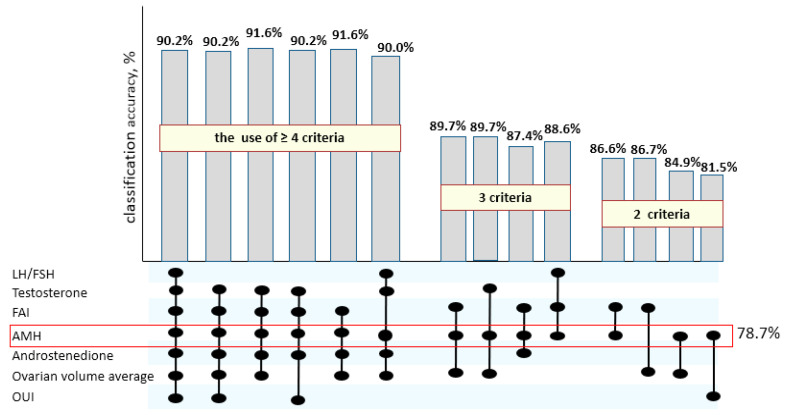
The diagnostic accuracy of the identified cutoff values of hormonal and ultrasound parameters in PCOS determination in adolescents. LH: luteinizing hormone; FSH: follicle-stimulating hormone; FAI: free androgen index; AMH: anti-Mullerian hormone; OUI: Ovarian to uterine index.

With the separate use of established cutoff values for AMH, FAI, androstenedione, LH/FSH, T, OV, and OUI as independent variables, a logistic regression model was significant but less accurate in PCOS prediction than with the combinations of detected hallmarks (Figure 2).

Thus, as can be seen from Figure 2, the combined use of four and more of suggested criteria allows one to determine PCOS in adolescents with the highest accuracy above 90%; the use of three parameters gives 85% of diagnostic precision, and it decreases when two or fewer criteria are used.

Moreover, the single use of AMH level, which indirectly characterizes ovarian morphology, determines PCOS prediction up to 78.7%, though it is not quite sufficient. The simultaneous estimation of the AMH level and the leptin concentration, which characterizes the metabolic features of the patients, allows one to reach the maximum classification accuracy of 91.8% already on the basis of two parameters (χ^2^(2) = 58.3, *p* < 0.00001). The results of performed logistic regression analysis define the formula for the PCOS prediction:

logit = 85.73−1.73 × (AMH)−0.12 × (Leptin),(3)

When the value of the function is less than 70.72, the diagnostic sensitivity is 92.5% and the specificity is 90.0%. Therefore, the measurement of concentrations of AMH and leptin in blood serum could be recommended for the determination of PCOS in adolescents with high accuracy.

## 4. Discussion

Diagnostic criteria of PCOS in adolescents remain controversial and are still under discussion [5,8]. In 2003, a group of experts from the European Society for Human Reproduction and Embryology (ESHRE) and the American Society for Reproductive Medicine (ASRM) in Rotterdam formulated the most widely used criteria for PCOS diagnostics based on the presence of two of the following indications: oligo-/anovulation (ANO), clinical and/or biochemical HA, and sonographic signs of polycystic ovaries (PCO) [3]. At the same time, it is generally accepted that ultrasound PCO morphology without HA or menstrual irregularities should not be used to diagnose PCOS in teenagers [6]. Therefore, Rotterdam criteria suggest that all three elements should be present for diagnosis of PCOS in adolescents [12,13]. The Endocrine Society and the Pediatric Endocrine Society clinical guidelines recommend use of criteria of National Institute of Health (NIH) for diagnosis of PCOS in adolescents in the presence of HA and persistent oligo-anovulatory menstrual cycles [13].

However, clear thresholds to identify hyperandrogenic state in adolescents are not established, and the data about normal testosterone fluctuations during adolescence are limited. Besides, the testosterone assays vary widely depending on the laboratory equipment and reagents.

Our study of hormonal profile analysis of PCOS patients compared to the control group revealed a significant increase in the level of LH, the ratio of LH/FSH, T, FAI, 17-OH-progesterone, DHEAS, androstenedione, and AMH, which corroborate the data of other studies [3,6]. Similar to the data of Güdücüa et al., our results showed positive correlations of FAI with BMI, WC/HC, and VAI, which were not significant in the group of “healthy” girls [14]. These observations confirm the positive association of ovarian HA with atherogenic blood profile and the risk of cardiovascular complications in patients with PCOS, even in adolescence.

One of the significant PCOS hallmarks interrelated with insulin resistance and HA is a decreased SHBG level [4,13,14]. In our study, we found significantly reduced SHBG level in patients with PCOS compared to the control group (37.0 nmol/L (24.7–55.5) vs. 52.9 nmol/L (39.0–67.6); *p* < 0.05), which determined a higher level of FAI (5.5 (2.8–7.0) vs. 1.6 (1.1–2.3); *p* < 0.0001). The study of Yetim et al. (2016) involving 53 patients with PCOS at the age of 15–20 and 26 controls showed significantly lower median of SHBG level in PCOS group (25.8 nmol/L vs. 49.6 nmol/L; *p* < 0.01) and relevantly higher median of FAI (6.8 vs. 3.0; *p* < 0.0001), which is comparable with our data [2].

In the systematic review, Tsikuras et al. (2015) emphasize the difficulty in diagnosing PCOS in adolescence not only due to the heterogeneous clinical picture but also due to the lack of clear values of hormonal parameters and HA in adolescents [3]. The authors summarized the data from 24 studies and revealed that adolescents with PCOS compared to controls were characterized with slightly higher levels of testosterone, DHEAS, LH, LH/FSH, and androstenedione in half of the cases and often corresponded to reference intervals. Focusing on our results, the levels of AMH (75.4%), LH (62.3%), and androstenedione (66.2%) were elevated over laboratory references in the majority of patients with PCOS; consequently, these parameters were the most significant in the diagnostics. Other indicators differed from the standards in less than half and even in one third of cases. Thus, based on hormonal parameters in comparison with the existing reference intervals for adolescents, the probability of correct prediction of the diagnosis is not high enough.

Most authors underline the importance of FAI and androstenedione estimation in HA assessment in adolescence [3,8,13]. The results of Pinola et al. showed that the androgen level in PCOS decreases with age [9]. Besides, they demonstrated that the levels of androstenedione > 9.65 ng/mL and FAI > 2.34 had high sensitivity and specificity in the diagnosis of PCOS in women under 40 years old [9]. Based on the results of our study, the values of FAI > 2.75, androstenedione > 11.45 ng/mL, and LH/FSH ratio > 1.23 could be used for PCOS diagnostics in this age interval with a high sensitivity of 63.2–78.2% and a specificity of 84.4–93.7%. Despite the obvious relevance, such data in adolescents are quite limited in the available literature.

In 2015, the World Pediatric Consensus (PedC) suggested the possibility of using the AMH level as a non-invasive screening test for PCOS diagnosis [11]. Yetim et al. calculated cut-off for the AMH for 15–20 years old youth with PCOS and showed that AMH value > 6.10 ng/mL had 92.3% sensitivity and 81.1% specificity in the diagnosis [2]. The research based on examining of 102 adolescents with PCOS, conducted by Merino et al., recommended the threshold for AMH > 7.03 ng/mL with 50.0% sensitivity and 70.8% specificity [11]. Though such investigations in adolescence are rare, according to our results, the use of AMH levels > 7.20 ng/mL with high sensitivity and specificity (75.0% and 89.0%) could be one of the perspective diagnostics tests of PCOS in girls at the age of 15–17 years.

On the other hand, using only the AMH level does not provide a sufficiently high accuracy in the diagnosis of PCOS. According to our study, the combined use of four or more from detected hormonal and ultrasound parameters (AMH, FAI, androstenedione, LH/FSH, T, OV, and OUI) is significantly more effective in PCOS prediction in adolescents than the separate use of each of them. Besides, we found that the high PCOS prediction up to 92% could be based on two parameters by adding leptin to the AMH level with 92.5% sensitivity and 90.0% specificity. Hence, the measurement of AMH and leptin concentrations in serum could be beneficial for PCOS diagnosis in adolescents.

According to our study, the leptin level was significantly higher in PCOS adolescents than in controls and was associated with BMI, WC, HC, and insulin level. It is well known that leptin is a proinflammatory cytokine secreted mainly by white adipose tissue, which plays an essential role in the regulation of food intake and energy expenditure [15,16,17]. In PCOS patients, high leptin concentrations are associated with insulin resistance and glucose intolerance, thus it could be used as indicator of metabolic disorders. Besides, leptin and insulin resistance increase ovarian sensitivity to LH and contribute to hyperandrogenism progression and anovulation in PCOS patients. In the research, Daan NM et al. showed high correlation between leptin and adiponectin with FAI in women with PCOS [15]. The results of Dumestic A et al. confirmed the pathogenic influence of metabolic disorders in HA progression in patients with PCOS [17]. The authors revealed that even normal-weight women with PCOS compared to normal-weight women without PCOS were characterized with increased adipose-insulin resistance and altered subcutaneous abdominal adipose stem cell gene expression, which correlated with hyperandrogenemia and the whole-body IR. In our study, adolescent girls with PCOS did not differ in terms of lipid profile from normal-weight healthy girls, except in the parameters of BMI, WC/HC, and visceral adiposity index. However, when analyzing separately normal and overweight patients with PCOS, we found differences not only in dyslipidemia and atherogenic lipid profile but also in more pronounced hyperandrogenism according to the increased level of FAI and decreased SHBG in overweight PCOS group. Besides, our findings corroborate with our previous results in which high leptin and CRP levels were associated with activated oxidative stress and systemic inflammation in overweight adolescents with PCOS complicated with metabolic disorders [18]. One of the generally accepted diagnostic criteria for PCOS is polycystic ovarian morphology according to ultrasound, though its evaluation in adolescence is doubtful [13]. The research by Grigorenko et al. showed that the transrectal access in girls allows better visualization of the ovaries and the follicular features compared to the transabdominal [19]. The examination of the 83 patients with PCOS at the age of 14–16 years showed increased ovarian volume, dilatation of arcuate veins and uterine plexus vessels, and hypervascularization of ovarian stroma about two times more frequently in girls with PCOS compared to healthy girls. Taking into account the size and the location of follicles, the authors identified the equal-caliber (57%) and the different-caliber (43%) ovarian morphology, which were associated with different hormonal levels in adolescents with PCOS. However, 3D transrectal visualizing requires expert class equipment and is not generally available. The 2D ultrasound results of our study showed high sensitivity of 81.0–83.0% and specificity of 83.0% of OV average > 10.70 cm^3^ and OUI > 3.9, which could be recommended as a screening method for examining adolescents at risk for PCOS.

Thus, our results are consistent with the recent studies and emphasize the importance of hormonal thresholds evaluation for PCOS determination in adolescents. To develop early recognition and prevent long-term reproductive, cardio-vascular, and psycho-emotional complications, adolescents with persistent HA and oligo-anovulation should be assessed for PCOS. The defined cut-off values for AMH, FAI, androstenedione, testosterone, and LH/FSH could be helpful to diagnose PCOS in adolescents with a high accuracy.

## 5. Conclusions

1. The risk of PCOS development is 4.9 times higher with the presence of gynecological pathology in a patient’s mother (95% CI = 1.33; 18.53, *p* = 0.0157).
2. Adolescents with PCOS in comparison with “healthy” girls are characterized by higher BMI [OR 1.31 (95% CI 1.10–1.56), *p* = 0.0021] and WC [OR 1.11 (95% CI 1.01–1.19), *p* = 0.0237] as well as a higher cardio-metabolic risk according to the visceral adiposity index (*p* = 0.0461).
3. Ultrasonographic parameters of average ovarian volume> 10.70 cm^3^ and ovarian to uterine index > 3.95 were highly sensitive (81.0–83.0%) and specific (83.0%) in PCOS determining in adolescents aged 15–17 according to 2D ultrasound screening.
4. The combined use of either four evaluated thresholds for AMH > 7.20 ng/mL, FAI > 2.75 ng/mL, testosterone > 1.15 nmol/L, androstenedione > 11.45 ng/mL, and LH/FSH ratio > 1.23 showed diagnostic accuracy above 90% in PCOS predicting in adolescents.

The accuracy of PCOS diagnostics in adolescents reached 92% using AMH and leptin concentrations when the value of the logistic regression function [85.73 − (1.73 × AMH) − (0.12 × Leptin)] was less than 70.72.

## Figures and Tables

**Table 1:** Blood biochemical analysis, lipid profile values, results of oral glucose tolerance test (OGTT) and Visceral Adiposity Index (VAI) values in adolescents with polycystic ovary syndrome (PCOS) and “healthy” girls.

Parameters	PCOS Group (*n* = 130)	Control Group (*n* = 30)	*p*-Level
Protein total *, g/L	72.2 (69.7–76.1)	70.6 (69.7–72.1)	0.0362
Urea *, mmol/L	4.0 (3.2–4.7)	4.5 (3.4–4.6)	0.7195
Creatinine *, μmol/L	75.6 (68.4–84.6)	74.4 (67.0–83.4)	0.9941
Bilirubin total, μmol/L	12.2 (9.5–18.2)	10.6 (8.3–14.0)	0.0789
Bilirubin direct, μmol/L	4.0 (2.7–5.2)	2.9 (2.4–3.8)	0.0289
Calcium *, mmol/L	2.4 (2.3–2.5)	2.4 (2.3–2.4)	0.4003
Magnesium *, mmol/L	0.8 (0.7–0.8)	0.6 (0.6–0.7)	0.0054
Iron *, μmol/L	18.0 (11.6–24.3)	15.6 (10.9–19.6)	0.2107
Cholesterol *, mmol/L	4.3 (3.9–5.0)	3.9 (3.5–4.7)	0.2511
TG *, mmol/L	0.8 (0.6–1.2)	0.7 (0.7–0.9)	0.3366
HDL *, mmol/L	1.4 (1.2–1.6)	1.6 (1.2–1.9)	0.0813
LDL *, mmol/L	2.1 (1.7–2.7)	1.9 (1.6–2.4)	0.2716
Atherogenic index *	2.1 (1.6–2.8)	1.7 (1.4–1.9)	0.0513
Glucose 0’ *, mmol/L	5.0 (4.7–5.3)	5.0 (4.6–5.2)	0.5548
Glucose 120’ *, mmol/L	6.0 (5.3–6.9)	–	–
Insulin 0’ *, mIU/ml	13.3 (8.7–19.3)	11.0 (8.2–11.9)	0.2306
Insulin 120’ *, mIU/ml	30.4 (14.4–47.4)	–	–
НОМА–IR *	2.9 (1.8–4.1)	2.3 (1.9–2.8)	0.2886
VAI *	1.0 (0.6–1.6)	0.7 (0.5–1.0)	0.0461
CRP *, mg/L	0.6 (0.2–1.3)	0.8 (0.3–1.3)	0.4772

* variable with a non-normal distribution, data are presented as median (25–75 percentiles), Mann–Whitney U-test. TG: triglycerides; LDL: low-density lipoproteins; HDL: high-density lipoproteins; HOMA-IR: Homeostasis Model Assessment of Insulin Resistance; CRP: highly sensitive C-reactive protein.

**Table 2:** Hormonal blood profile in adolescents with PCOS and “healthy” girls.

Parameters	PCOS Group (*n* = 130)	Control Group (*n* = 30)	*p*-Level
LH **, IU/L	9.0 (5.4–13.8)	3.7 (2.5–4.7)	<0.0001
FSH **, IU/L	5.6 (4.4–6.9)	5.3 (4.1–6.5)	0.2856
LH/FSH **	1.6 (1.0–2.3)	0.7 (0.5–1.1)	<0.0001
Testosterone **, nmol/L	1.9 (1.2–2.5)	0.8 (0.7–1.1)	<0.0001
SHBG **, nmol/L	37.0 (24.7–55.5)	52.9 (39.0–67.6)	0.0136
FAI **	5.5 (2.8–7.0)	1.6 (1.1–2.3)	<0.0001
Cortisol *, nmol/L	464.0 ± 147.6	284.0 ± 129.7	<0.0001
Prolactin **, mlU/L	266.0 (175.0–405.0)	189.0 (142.0–269.0)	0.0141
17-OHP **, nmol/L	4.1 (3.2–5.1)	3.4 (2.7–3.8)	0.0071
DHEAS *, μmol/L	6.8 ± 3.2	5.1 ± 1.5	0.0039
DHEAS/FAI **	1,3 (0,9–2,1)	3,2 (1,8–5,7)	<0,0001
Androstenedione **, ng/mL	15.8 (11.6–23.2)	8.3 (6.5–10.8)	<0.0001
АМH **, ng/mL	9.5 (7.5–14.9)	5.8 (3.8–6.9)	<0.0001
Leptin *, ng/mL	38.7 ± 27.1	23.7 ± 14.0	0.0178

* variable with a normal distribution, data are presented as mean ± standard deviation, Student’s t-test. ** variable with a non-normal distribution, data are presented as median (25–75 percentiles), Mann–Whitney U-test. LH: luteinizing hormone; TSH: thyroid-stimulating hormone; FSH: follicle-stimulating hormone; SHGB: sex hormone binding globulin; FAI: free androgen index; 17-OHP: 17α-OH progesterone; DHEAS: dehydroepiandrosterone sulfate; AMH: anti-Mullerian hormone.

**Table 3:** Ultrasound features of uterus and ovaries in adolescents with PCOS and “healthy” girls.

Parameters	PCOS Group (*n* = 130)	Control Group (*n* = 30)	*p*-Level
Uterus length *, cm	4.4 ± 0.7	4.4 ± 0.5	0.7553
Uterus thickness *, cm	2.9 ± 0.5	2.8 ± 0.5	0.2862
Uterus width *, cm	4.3 ± 0.6	4.3 ± 0.5	0.7571
Endometrium **, cm	0.5 (0.4–0.7)	0.3 (0.2–0.4)	<0.0001
Uterine volume *, cm^3^	17.6 ± 8.4	16.5 ± 7.2	0.4653
Cervix length **, cm	2.7 (2.3–3.1)	3.2 (3.0–3.6)	0.0005
Ovarian length *, cm	4.0 ± 0.7	3.6 ± 0.7	0.0093
Ovarian thickness *, cm	2.4 ± 0.6	1.7 ± 0.3	<0.0001
Ovarian width *, cm	2.8 ± 0.8	2.3 ± 0.5	0.0010
The average ovarian volume (OV), cm^3^	14.5 ± 6.7	8.2 ± 3.7	<0.0001
Ovarian to uterine index (OUI)	4.9 (3.6–6.5)	3.3 (1.9–3.8)	<0.0001
The number of follicles in the slice, n	14.0 (12.0–15.0)	8.0 (6.8–9.0)	<0.0001
The diameter of the minimum follicle, mm	3.0 (2.0–5.0)	5.0 (4.0–6.0)	<0.0001
The diameter of the maximum follicle, mm	7.0 (5.0–8.0)	8.0 (7.0–9.0)	0.0034

* variable with a normal distribution, data are presented as mean ± standard deviation, Student’s t-test. ** variable with a non-normal distribution, data are presented as median (25-75 percentiles), Mann–Whitney U-test.
